# The Circadian Hormone Melatonin Inhibits Morphine-Induced Tolerance and Inflammation via the Activation of Antioxidative Enzymes

**DOI:** 10.3390/antiox9090780

**Published:** 2020-08-22

**Authors:** Ing-Jung Chen, Chih-Ping Yang, Sheng-Hsiung Lin, Chang-Mei Lai, Chih-Shung Wong

**Affiliations:** 1 Department of Anesthesiology, Cathay General Hospital, Taipei 10630, Taiwan; dr.jungchen@gmail.com; 2 Department of Medical Research, Cathay General Hospital, Taipei 10630, Taiwan; 3 Department of Anesthesiology, Chi-Mei Medical Center, Tainan 71004, Taiwan; ycp810@gmail.com; 4 Department of Anesthesiology, School of Medicine, National Defense Medical Center, Taipei 11490, Taiwan; 5 Planning & Management Office, Tri-Service General Hospital, Taipei 11490, Taiwan; linbill600328@gmail.com; 6 Graduate Institute of Medical Sciences, National Defense Medical Center, Taipei 11490, Taiwan; iem181711@gmail.com

**Keywords:** melatonin, circadian rhythms, morphine tolerance, neuropathic pain, chronic constriction injury, antioxidants, antioxidative enzyme, *Kcnip3*, DREAM

## Abstract

Opioids are commonly prescribed for clinical pain management; however, dose-escalation, tolerance, dependence, and addiction limit their usability for long-term chronic pain. The associated poor sleep pattern alters the circadian neurobiology, and further compromises the pain management. Here, we aim to determine the correlation between constant light exposure and morphine tolerance and explore the potential of melatonin as an adjuvant of morphine for neuropathic pain treatment. Methods: Wistar rats were preconditioned under constant light (LL) or a regular light/dark (LD) cycle before neuropathic pain induction by chronic constriction injury. An intrathecal (i.t.) osmotic pump was used for continued drug delivery to induce morphine tolerance. Pain assessments, including the plantar test, static weight-bearing symmetry, and tail-flick latency, were used to determine the impact of the light disruption or exogenous melatonin on the morphine tolerance progression. Results: constant light exposure significantly aggravates morphine tolerance in neuropathic rats. Continued infusion of low-dose melatonin (3 μg/h) attenuated morphine tolerance in both neuropathic and naïve rats. This protective effect was independent of melatonin receptors, as shown by the neutral effect of melatonin receptors inhibitors. The transcriptional profiling demonstrated a significant enhancement of proinflammatory and pain-related receptor genes in morphine-tolerant rats. In contrast, this transcriptional pattern was abolished by melatonin coinfusion along with the upregulation of the *Kcnip3* gene. Moreover, melatonin increased the antioxidative enzymes SOD2, HO-1, and GPx1 in the spinal cord of morphine-tolerant rats. Conclusion: Dysregulated circadian light exposure significantly compromises the efficacy of morphine’s antinociceptive effect, while the cotreatment with melatonin attenuates morphine tolerance/hyperalgesia development. Our results suggest the potential of melatonin as an adjuvant of morphine in clinical pain management, particularly in patients who need long-term opioid treatment.

## 1. Introduction

Opioids are important and effective analgesics for the management of peri/postoperative pain and cancer pain. They are also commonly prescribed as long-term management for chronic noncancer pain [1]; however, adverse consequences are associated, such as tolerance, hyperalgesia, or even addiction [2,3]. In the early 2000s, prescribed opioids for chronic pain increased in the United States (from approximately 100 to approximately 700 morphine milligram equivalents per person per year) and was accompanied by increasing unintended overdoses and associated deaths [4,5]. In response to this opioid epidemic, interventions including patient risk assessment, psychotherapy, the patient-centered taper of opioid dosage, and medication-assisted treatment have been proposed for the identification of patients at risk and to prevent those patients from falling into overdose and addiction [6].

Patients who suffered from chronic pain also tend to have a higher prevalence of sleep disorders (up to 44%), which mainly manifests as impaired sleep initiation and maintenance [7]. Moreover, the disrupted central/peripheral circadian rhythms could lead to oxidative stress, neuroinflammation, and eventually neurodegenerative diseases [8]. Circadian rhythms and their correlation with the efficacy of antinociceptive drugs have gained attention in recent years [9,10]. Morris et al. first reported a maximum antinociceptive effect of intraperitoneally injected (i.p.) morphine under dark conditions (21:00 h) in a mouse model [11]. Next, Kavaliers et al. also demonstrated that the change of daily rhythms alters the analgesia of morphine in mice, with an enhanced analgesic effect in the dark cycle [12]. A clinical retrospective study observed a lower demand for self-administered morphine in adults with cancer pain at night [13]. Furthermore, morphine showed considerable pharmacokinetic variation depending on the time of injection during the day [14]. A circadian pattern on morphine analgesia implies the regulation of chronobiology in pain modulation, as well as the neuronal sensitivity to the drug.

Melatonin is a neurohormone synthesized and secreted mainly by the pineal gland [15]. In both rodents and humans, melatonin reaches its peak secretion at night and light exposure suppresses its production and secretion [16,17,18]. In humans, melatonin circulates in both plasma and cerebrospinal fluids (CSF) with a nocturnal peak concentration of 122–660 pmol/L and 94–355 pmol/L, respectively [19]. Similarly, Wistar rats have peak plasma and pineal gland levels of melatonin at midnight (00:00–03:00, 12 h light/12 h dark cycle with lights on at 06:00 h) [16]. This circadian oscillation of melatonin in serum and urine has made it a biomarker for disrupted circadian rhythm patterns and correlated sleep disorders [20]. Moreover, melatonin plays a significant role in the regulation of neuropathic pain based on rodent model experiments, showing remarkable analgesic potency [21]. For instance, Borsani et al. showed a single dose of melatonin (i.p.,10 mg/kg) rescued thermal hypersensitivity for up to 3 h on day 14 in chronic constriction injury (CCI)-induced neuropathic rats [22]. Zurowski et al. also demonstrated that 100 mg/kg i.p. of melatonin abolished mechanical allodynia in CCI neuropathic rats [23]. Moreover, Huang et al. found that a daily dose of melatonin (75–300 mg/kg) alleviates hypersensitivity, even in sleep-deprived neuropathic rats [24]. However, the analgesic doses of melatonin given to rodents (up to 200 mg/kg i.p. or 300 mg/kg by oral administration (p.o.)) were higher than the physiological level and the conventional human dosage for promoting sleep in clinical practices (0.1 mg–10 mg per day, p.o.) [25,26,27]. Also, a previous human interventional study found that a high dose of melatonin (10 or 100 mg via intravenous injection (i.v.)) could not provide a direct analgesic or antihyperalgesic effect on the experimental human burn injury [28]. Nevertheless, several studies have demonstrated that melatonin (3–10 mg, given before surgery) reduces preoperative anxiety and postoperative pain while improving the patient’s sleep quality [29].

The differential findings suggested the importance of drug dose, timing, and the interaction of melatonin with anesthetics. The potential cotreatment of melatonin with morphine has been explored in multiple scenarios [30]. Raghavendra and Kulkarni first showed the role of melatonin in the progression of morphine tolerance development [31]. They reported the reversal of morphine-induced tolerance in mice via systemic coadministrated melatonin (1–10 mg/kg, i.p.) [32]. Zahn et al. found that 100 nmol melatonin administered intrathecally (i.t.) caused a brief antinociceptive effect when cotreated with a low and nonresponse dose (0.1 μg, i.t.) of morphine [33]. A similar morphine enhancer effect was reported in mice at a dose of 20 mg/kg melatonin using a formalin-induced pain model [34]. Next, Garmabi et al. found a reduction of melatonin levels in rats under constant light exposure; those animals presented higher morphine consumption and more severe morphine withdrawal syndrome [35]. Moreover, Fan and Song et al. reported a significant decrease in serum melatonin and MT1 receptor mRNA after chronic morphine infusion in rats [36]. Song et al. also concluded that daily administration of melatonin (10 mg/kg i.p.) reduces morphine tolerance in rats via inhibition of the N-methyl-D-aspartate (NMDA) receptor’s NR1 subunit expression [37]. Recently, we found that an i.t. injection of melatonin (50 μg) rescued the morphine antinociceptive effect via inhibiting microglial activation and heat shock protein 27 expression [38]. Our microarray analysis also showed that a single i.t. injection of melatonin in the morphine-tolerant rat inverts the bulk of genes down/upregulated by the chronic morphine treatment [39], suggesting a crucial genetic regulation of high-dose melatonin treatment and consequently the morphine tolerance reversal. However, the effect of long-term administration of melatonin directly targeting the spinal cord on a morphine-tolerant rat model has not been fully explored. Our i.t. drug delivery model could further explore the potential morphine enhancer effect of this circadian hormone in the central nervous system. In this study, we aim to explore the pain-behavior influence of constant light exposure and chronic melatonin infusion on neuropathic pain management with morphine infusion. Also, we examine the mechanistic involvement using transcriptional analysis and determine the impact on antioxidative defense.

## 2. Materials and Methods

### 2.1. Reagents

Both morphine sulfate and melatonin were purchased from Sigma-Aldrich (Sigma-Aldrich, St. Louis, MO, USA), and the melatonin metabolite AFMK (*N*-Acetyl-*N*-formyl-5-methoxykynurenamine) was purchased from Cayman Chemical (Ann Arbor, MI, USA). Melatonin and AFMK were freshly prepared before the infusion experiment. The powders were first dissolved (100 mg/mL) in the dimethyl sulfoxide (DMSO) solvent (Sigma-Aldrich, St. Louis, MO, USA), then diluted with 0.9% saline or morphine solution into the final working concentration (3 μg/μL). The saline-DMSO and MOR(morphine)-DMSO infusion pump also contained an equal amount of DMSO, ruling out the solvent effect.

### 2.2. Experimental Design

All animal care and experimental protocols complied with institutional and international standards and were approved by the Institutional Animal Care and Use Committee (IACUC, Approval no. 107-002) of Cathay General Hospital (Taipei, Taiwan). Adult male Wistar rats were purchased from BioLASCO Taiwan Co., Ltd. (Yilan, Taiwan) and housed in the Cathy Medical Research Center at a temperature of 22 ± 2 °C with 55% humidity, with free access to the standard chow diet and water. As described in previous reports [40,41], morphine tolerance was induced by continued infusion via an i.t. catheter coupled with an osmotic pump.

In the naïve rat model, Wistar rats were housed under a regular 12/12 h light/dark cycle. Before i.t. pump placement, the tail-flick test was done as the baseline and followed up on the first and 5th days after morphine infusion (i.t., 15 μg/h). On the fifth day, a single bolus of morphine (15 μg/5 μL, i.t.) was injected to validate the morphine tolerance based on the tail-flick response. Either melatonin (3, 6, or 12 μg/h) or saline solvent was coadministered with morphine (i.t.) infusion to evaluate the long-term effect of melatonin.

To establish a circadian disruption, Wistar rats were randomly relocated to a regular 12/12 h light/dark (LD) cycle or constant light (LL) room for two weeks. After the preconditioning with distinct light exposure, rats were subjected to chronic constriction injury (CCI) surgery on the left sciatic nerve to induce neuropathic pain. Then, rats were maintained respectively in their LD or LL room, and the pain on the left paw was evaluated by both plantar and weight-bearing tests. The morphine tolerance was induced on the 4th day via i.t. morphine pump (15 μg/h) and followed up for an additional six days. The noninfused CCI rats of both LL and LD cycles served as the non-morphine-tolerance controls, and the sham-operated rats as the non-neuropathic controls were placed in the same LD or LL room.

In the neuropathic pain model, Wistar rats were subjected to CCI surgery on the left sciatic nerve to induce neuropathic pain. Three days after, pain on the left paw of the rats was evaluated by the plantar and weight-bearing tests as validation of the established neuropathic pain. Then, an i.t. pump was placed for the continued delivery of morphine alone (15 μg/h) or combined with melatonin (3 μg/h) on the 4th day post-CCI surgery. The neuropathic pain was followed up using the plantar and weight-bearing tests on days 5, 7, and 10 post-CCI surgery. The CCI rats with saline pump infusion served as the non-morphine-tolerance control.

### 2.3. Chronic Constriction Injury

The unilateral sciatic nerve constriction injury proceeded with minor modifications, as described in the previous report [42]. Briefly, male Wistar rats (300–350 g) were anesthetized with 2% isoflurane. With the rat lying on its chest, the left hind leg was held in position with the femur at 90° to the spine on the surgical table; the operation area was shaved and sterilized with 70% isopropyl alcohol and iodine solution. An incision on the skin was made at 3–4 mm below the femur. The connective tissue between the gluteus superficialis and the biceps femoris muscles was freed with blunt scissors. A retractor was placed to widen the gap between the two muscles, allowing clear visualization of the left sciatic nerve. Approximately 10 mm of the sciatic nerve (proximal to the sciatic trifurcation) was freed from the surrounding connective tissue. Three ligatures (chromic gut 4.0) were tied with a double knot, with 1 mm space between the ligature and proximal to the trifurcation of the sciatic nerve. Then, the muscle layer was closed with sutures (chromic gut 4.0), and the skin fastened with Nylon 3.0 sutures. Following surgery, 100 mg/kg of cefazolin was administrated for the prevention of infection. The sham operation repeated all the procedures described above, except the ligatures of the sciatic nerves, and served as the non-neuropathic control. All rats were carefully observed during the anesthesia recovery period and returned to their cage under a conscious state. Rats with motor deficits or excessive behavior stress were excluded.

### 2.4. Intrathecal Drug Delivery

Insertion of the i.t. catheter was performed as described in previous reports [40,43]. Wistar rats (350–400 g) were anesthetized with pentobarbital (65 mg/kg, i.p.). The two catheters went from the atlantooccipital membrane opening down to the lumbar enlargement (L1–L2) of the spinal bony structure. Intrathecal catheters were constructed by using polyethylene tube (8.5 ± 0.5 cm in length, 0.008 in. inner diameter and 0.014 in. outer diameter) and a 3.5 cm Silastic^®^ tube. The outer end of one catheter (for morphine challenge) was fixed to the dorsal aspect of the head. The other catheter was connected to a subcutaneous miniosmotic pump (delivering 1 μL per hour; Alzet, Cupertino, CA, USA) and used for intrathecal drug infusion according to the experimental design. After catheterization, 100 mg/kg of cefazolin was administered to prevent infection. Rats with neurological deficits after the procedure were excluded.

### 2.5. Nociceptive Test

(1) Tail-flick latency was examined using the hot water immersion method (52 ± 0.5 °C). Rats were placed in plastic restrainers for tail-flick assessment, and a maximum of 10 s was set as the cutoff time in order to avoid tail injury. (2) The plantar test measured the paw withdrawal threshold of the animal using a Dynamic Plantar Aesthesiometer (Ugo Basile, Gemoio, Italy). Rats were placed on the testing platform, and the touch stimulator with a filament actuator was set at maximum force of 50 g in 25 s. The force actuator automatically detected and recorded the actual force at the time of paw withdrawal as a response of allodynia. (3) Weight-bearing test: Hind paw static weight-bearing was measured using an incapacitance tester (Linton Instrumentation, Norfolk, UK) to detect CCI-induced changes in weight-bearing symmetry. The rats were placed on their hind paws in a box containing an inclined plane (65° from horizontal) that was placed above the incapacitance apparatus. The weight that the animals applied to each hind limb was measured independently by the apparatus. Five measurements were taken and averaged for each rat. The data are expressed as the difference between the weight applied to the naïve hind limb (right) and the weight applied to the CCI-operated side (left) (Δ force, g). An increased Δ force suggests a perceived pain due to the CCI operation on the left side [44].

### 2.6. Cell Culture

Mouse microglia ECO 13.31 cells were purchased from the Bioresource Collection and Research Centre (Hsinchu, Taiwan). They were grown in Dulbecco’s modified Eagle’s medium with 4 mM L-glutamine adjusted to contain 1.5 g/L sodium bicarbonate and 4.5 g/L glucose, 70%; fetal bovine serum, 10%; and LADMAC Conditioned Media (produced from the LADMAC cell line), 20%. Cultures were maintained at 37 °C with 5% CO_2_, and the media was changed every 2–3 days. Before drug administration, microglia cells were harvested, counted, and resuspended in growth media at 5 × 10^5^ cells/well in a 6-well plate for treatment.

### 2.7. Spinal Cord Sample Preparation

All rats were sacrificed at the end of the final behavior test by exsanguinations under isoflurane-induced anesthesia. Laminectomy was performed at the lower edge of the 12th thoracic vertebra, and the L5–S3 segment of the spinal cord was removed immediately and separated into the ventral and dorsal parts. The dorsal portion of the spinal cord was used for total RNA isolation and protein preparation.

### 2.8. Western Blot Analysis

The spinal cord dorsal horn and ECO 13.31 cells were homogenized in ice-cold radioimmunoprecipitation assay buffer (Bio-Rad, Hercules, CA, USA) containing a protease inhibitor cocktail (MedChemExpress, Monmouth Junction, NJ, USA). The lysate was centrifuged at 12,000× *g* for 30 min at 4 °C, and the supernatant total protein lysates were used for Western blotting analysis. First, the protein concentration of the sample was determined by DC protein assay (Bio-Rad). An equal amount of total protein was adjusted to a similar volume with Laemmli sample buffer with 5% β-mercaptoethanol as a reducing agent and denatured by heating at 95 °C for 5 min. Samples were separated on 8–12% SDS-polyacrylamide gels and transferred onto polyvinylidene fluoride (PVDF) membranes (Millipore Corporation, Billerica, MA, USA). The membranes were blocked with blocking solution and incubated overnight at 4 °C with the following antibodies: NOX2, SOD2, HO-1, GPx1 (Abcam, Cambridge, MA, USA), and GAPDH (Proteintech Group, Rosemont, IL, USA) as a loading control. After three washes with PBST (1× PBS + Tween 20 0.1%) buffer, membranes were incubated for one hour at room temperature with appropriate horseradish peroxidase (HRP)-conjugated secondary antibodies. Luminescence signals were triggered using the chemiluminescence reagent ECL™ Prime (GE Healthcare Bio-Sciences, Pittsburgh, PA, USA) and visualized under an Alpha Innotech FluorChem FC2 Imaging System (Alpha Innotech Corp, San Leandro, CA, USA). The densities of each specific band were measured using ImageJ (version 1.52t).

### 2.9. RNA Extraction and Quantitative PCR

RNA was isolated from the spinal cord dorsal horn using Rezol (PROtech technologies, Taiwan). The cDNA was reverse-transcribed using random hexamers from 1.5 μg of total RNA using Maxima H minus Reverse Transcriptase (Thermo Scientific, Hemel Hempstead, UK) following the manufacturer’s instructions. RT^2^ Profiler™ PCR Array Rat Pain: Neuropathic and Inflammatory (Qiagen, Frederick, MD, USA) was carried out in the LightCycler^®^ 480 Instrument and raw data acquired with LightCycler^®^ 480 Software, version 1.5.0.39 (Roche Diagnostics GmbH, Germany).

### 2.10. Statistical Analysis

The data are expressed as the mean ± SEM. All graphical representations and statistical calculations were aided by GraphPad Prism version 6.01 and Microsoft Excel. The RT-PCR raw data were analyzed on Qiagen GeneGlobe web portal (http://www.qiagen.com/geneglobe), which calculated fold change/regulation using the 2^ (-delta delta CT) method. Two-way ANOVA, Tukey’s multiple comparisons test, Bonferroni’s multiple comparisons test, and Student’s t-test were used to analyze the statistical significance.

## 3. Results

### 3.1. Constant Light Exposure Aggravate Morphine Tolerance in Neuropathic Rats

**Figure 1:**
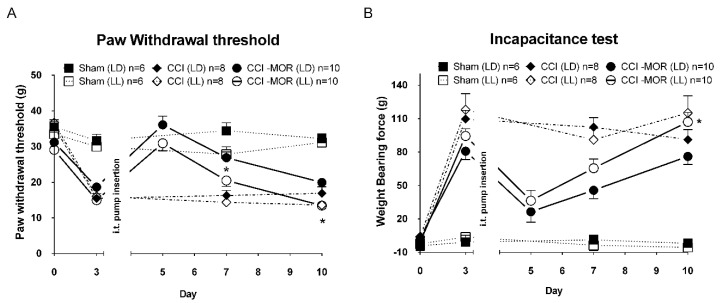
Circadian light disruption in rats with neuropathic pain. Rats were randomly preconditioned and housed in either a regular light/dark cycle (LD) or constant light (LL) room. The baseline paw withdrawal threshold (**A**) and weight-bearing test (**B**) were measured before chronic constriction injury of sciatic nerve (CCI) surgery and on day 3 post-CCI as the basal value before morphine treatment. Morphine (MOR) was infused (15 μg/h, i.t.) to CCI rats under both LD and LL cycles on day 4 (CCI-MOR(LD) and CCI-MOR(LL)) to induce tolerance. Pain behavior was assessed on days 5, 7, and 10 post-CCI surgery. The sham-operated rats under LD and LL circadian cycles served as non-neuropathic controls (Sham-LD and Sham-LL). * denotes a statistically significant difference between CCI + MOR(LD) vs CCI + MOR(LL); * *p* < 0.05.

Left chronic constriction injury of the sciatic nerve (CCI) was used to induce inflammatory/neuropathic pain in rats. Firstly, all rats were preconditioned under either constant light (LL) or a normal 12/12 h light/dark cycle (LD) for two weeks. As shown in Figure 1, under LL or LD conditions, the sham-operated rats showed no significant difference in either plantar test compared to the baseline on day 0. At regular LD cycles, CCI rats exhibited persistent allodynia, presenting a lower withdrawal threshold (Figure 1A, CCI (LD), 15.5 ± 1.3 g) and weight-bearing asymmetry (Figure 1B, CCI (LD), Δforce 109.5 ± 7.9 g) since the 3rd day after CCI surgery. The pain behavior lasted up to 10 days, validating our surgically induced neuropathic pain model. Overall, CCI rats living in the LL room manifested similar allodynia to CCI rats in the LD room.

Morphine tolerance was induced by continued intrathecal morphine infusion (15 μg/h), starting on day 4 post-CCI surgery in rats (CCI-MOR). CCI-MOR rats under both LL and LD cycles showed significant amelioration of pain on the 5th day due to morphine-induced antinociception, shown by restored plantar tests (Figure 1A, CCI-MOR(LD): 36.1 ± 2.4 g) and weight-bearing tests (Figure 1B, CCI-MOR(LD): Δforce 26.4 ± 9.3 g) compared to CCI only. However, from day 7 to 10 post-CCI surgery, the antinociceptive effects gradually decreased as the morphine tolerance developed in the CCI-MOR(LD) rats (Figure 1A, CCI-MOR(LD): 19.9 ± 1.1 g and Figure 1B, CCI-MOR(LD): Δforce 76.1 ± 7.3 g). Furthermore, the CCI rats under the LL condition showed significant hyperalgesia and an increased morphine tolerance on day 10 compared to CCI-MOR rats housed under an LD cycle (Figure 1A, CCI-MOR(LL):13.4 ± 0.7 g and Figure 1B, CCI-MOR(LL): Δforce 107.0 ± 8.5 g).

### 3.2. Exogenous Low-Dose Melatonin Inhibited Morphine Tolerance Independently of Melatonin Receptors

To find the optimal infusion dose, different doses of melatonin (1–6 μg/h, continued i.t. infusion) were cotreated with morphine-tolerant rats to examine its effect on morphine tolerance development (Figure S1); it showed that 3 μg/h melatonin infusion (3 μg/h Mela) preserved morphine’s antinociceptive effect, with higher tail-flick latency on day 5 than solvent-only (MOR + DMSO) morphine-tolerant rats. Surprisingly, a higher dose (6 μg/h) melatonin showed no benefit in comparison to morphine-tolerant rats (Figure S1A). Following the morphine challenge (15 μg/5 μL, i.t.), melatonin-treated (3 μg/h, i.t) rats showed the best morphine antinociception, compared to two other doses (Figure S1B).

**Figure 2:**
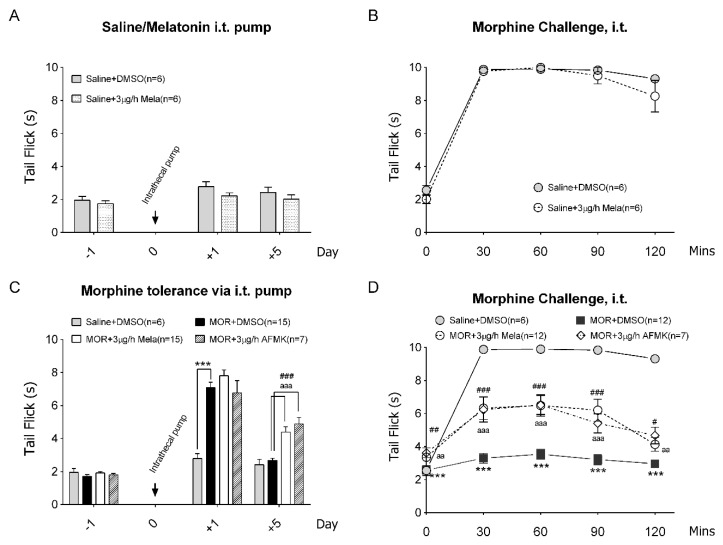
Low-dose melatonin coinfusion with morphine reduces morphine tolerance progression. (**A**) Continuous intrathecal melatonin (Mela, 3 μg/h) or solvent-DMSO infused via an osmotic pump. Antinociceptive tail-flick latencies (52 ℃ water) were measured 1 day before (−1), 1 day after (+1), and 5 days after (+5) after osmotic pump implantation (day 0). (**B**) After 5 days of i.t. pump infusion, rats were further challenged with 15 μg of morphine (i.t.), and the tail-flick test was performed every 30 min for 120 min. (**C**) Continuous administration of 3 μg/h Mela, melatonin metabolite *N*-Acetyl-*N*-formyl-5-methoxykynurenamine (AFMK), or solvent-DMSO in morphine (MOR)-induced tolerant rats. Tail-flick test was measured before and 1 and 5 days after pump implantation (day 0) for morphine tolerance measurement. (**D**) After 5 days of i.t. infusion, rats were further challenged with morphine (15 μg, i.t.), and the tail-flick test was performed for 120 min to validate the tolerance. * denotes statistically significant differences between saline + DMSO and MOR + DMSO, # denotes statistically significant differences between MOR + Mela and MOR + DMSO, and “a” denotes statistically significant differences between MOR + AFMK and MOR + DMSO: # *p* < 0.05; ##/aa *p* < 0.01; ###/***/aaa *p* < 0.001.

However, melatonin at 3 μg/h i.t. infusion did not produce any antinociceptive effect compared to the solvent control (saline + DMSO) from days 1 to 5 (Figure 2A) as measured by the tail-flick test. On day 5, both 3 μg/h Mela- and saline + DMSO-treated rats showed a remarkable antinociceptive effect following an i.t. morphine challenge, with an average 10 s latency at 30–90 min postinjection (Figure 2B). In the morphine-tolerant rats, we found significant antinociception on the first day as compared to the saline solvent control (Figure 2C, MOR + DMSO: 7.0 ± 0.3 s vs saline + DMSO: 2.8 ± 0.3 s). However, it was soon abolished on day 5 as morphine tolerance developed (Figure 2C, MOR + DMSO: 2.6 ± 0.1 s). In contrast, coinfused melatonin during morphine tolerance induction (MOR + 3 μg/h Mela) significantly preserved morphine’s antinociception at day 5 (Figure 2C, MOR + 3 μg/h Mela: 4.4 ± 0.3 s). Upon morphine challenge at day 5, melatonin-coinfused rats also showed a significant reversal of morphine sensitivity compared to solvent-treated morphine-tolerant rats (Figure 2D, at 60 min, MOR + 3 μg/h Mela: 6.5 ± 0.7 s vs MOR + DMSO: 3.5 ± 0.3 s). Next, we tested the effect of the melatonin metabolite *N*-Acetyl-*N*-formyl-5-methoxykynurenamine (AFMK), which is a potent free-radical scavenger independent of melatonin receptor action [45]. It showed that AFMK (3 μg/h, i.t.) treatment showed a similar effect to melatonin coinfusion (3 μg/h, i.t.) in morphine-tolerant rats (Figure 2C,D, MOR + 3 μg/h AFMK); this suggests that long-term low-dose melatonin i.t. infusion ameliorates morphine tolerance via a melatonin receptor-independent mechanism.

**Figure 3:**
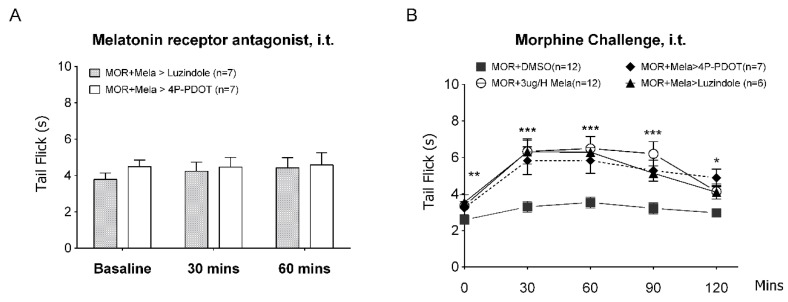
Melatonin protects morphine tolerance via an MT receptor-independent pathway. After five days of continuous melatonin administration (3 μg/h, i.t.) with morphine (15 μg/h) (MOR + Mela) infusion, the basal tail-flick was measured before (**A**) 10 μg of luzindole or 5 μg of 4P-PDOT i.t. injection for 60 min. (**B**) After luzindole or 4P-PDOT i.t. injection to MOR + Mela rats was further challenged with morphine (15 μg, i.t.), the tail-flick test was performed at 0–120 min to validate the morphine responsiveness. * denotes statistically significant differences between MOR + Mela and MOR + DMSO. * *p* < 0.05; ** *p* < 0.01; *** *p* < 0.001.

To further understand whether melatonin receptors are involved in the regulation of morphine tolerance, the MT receptor antagonist luzindole (a nonselective inhibitor, 10 μg) or 4P-PDOT (MT-2 selective inhibitor, 5 μg) was added on day 5 to MOR + Mela-infused rats. Surprisingly, we found that both inhibitors could not reverse the melatonin’s antitolerance effect on morphine tolerance, as shown in Figure 3. In MOR + Mela rats, we found no change of tail-flick latency after either luzindole or 4P-PDOT i.t. injection (Figure 3A). The inhibitor-treated rats still showed an antinociceptive response similar to MOR + 3 μg/h Mela-treated rats following morphine challenge (Figure 3B). Despite the presence of melatonin receptors antagonists, melatonin-cotreated rats still showed preserved morphine sensitivity compared to MOR + DMSO.

### 3.3. Melatonin Upregulated Antioxidative Enzymes and Decreased Proinflammatory Gene Expression in Morphine-Tolerant Rats

**Figure 4:**
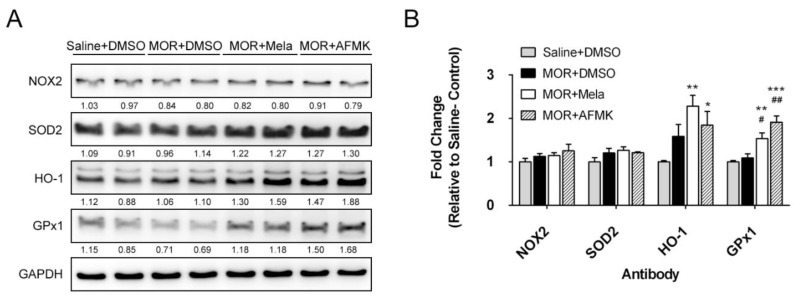
Melatonin cotreatment upregulated antioxidative enzymes in morphine-tolerant rat spinal cord. (**A**) Spinal cord (dorsal horn) total protein was collected from rats treated with saline alone, morphine (15 μg/h, MOR) alone, MOR combined with melatonin (3 μg/h) (MOR + Mela), or AFMK (3 μg/h) (MOR + AFMK). Relative protein levels of NOX2, SOD2, HO-1, GPx1, and GAPDH were determined by Western blot analysis. (**B**) Densitometry of Western blot. Each probe signal was normalized to GAPDH, and fold change was calculated relative to the saline control. Statistics were analyzed from four independent samples of each group. * denotes statistically significant differences compared to saline + DMSO. * *p* < 0.05; ** *p* < 0.01; *** *p* < 0.001. # denotes statistically significant differences compared to MOR + DMSO. # p < 0.05; ## *p* < 0.01.

As a further evaluation of the melatonin receptor-independent effect on morphine tolerance inhibition, the involvement of morphine in inflammation and oxidative stress [46] was evaluated via the expression of oxidative (NOX2) and antioxidative enzymes (SOD2, HO-1, and GPx1) in the spinal cords. As the representative Western blot shows in Figure 4A, the spinal NOX2 protein had a minor change in morphine-tolerant rats (Figure 4A, MOR). However, morphine tolerance did not change SOD2 and HO-1 levels, but GPx1 expression was reduced by as much as 31% compared to saline-treated rats. The 3 μg/h of melatonin coinfusion prevented reduction of the antioxidative enzyme GPx1, and further upregulated SOD2 (by up to 27%) and HO-1 (by up to 59%) expression in the spinal cord, without altering NOX2 expression (Figure 4A, MOR + Mela). Additionally, AFMK cotreatment (MOR + AFMK) showed a similar finding in the expression of the antioxidative enzymes SOD2 (by up to 30%), HO-1 (by up to 88%), and GPx1 (by up to 68%). Densitometry data showed that both melatonin and AFMK cotreatment significantly upregulated HO-1 expression compared to saline-DMSO, and significantly increased GPx1 protein expression compared to both saline and morphine alone (Figure 4B).

Microglial cells play an essential role in the oxidative status and antioxidative response in the central nervous system [47]. As shown in Figure S2, after 72 h, incubation of high-dose morphine (200 μM) downregulated both NOX2 and SOD2 expression, while the cotreatment with 50 μM melatonin rescued the NOX2 downregulation and further upregulated the expression of the SOD2 and HO-1 antioxidative enzymes (Figure S2).

We screened a panel of different genes’ expression based on the quantitative PCR method to evaluate the potential target genes modulated by melatonin in the morphine-tolerant rats (see the full table of gene expression analysis and full description of each gene in Tables S1 and S2, Supplementary Materials). The array contained genes related to the conduction of pain, synaptic transmission, and pain response modulation. Table 1 showed up to 12 mRNAs (*Ccr2*, *Il-6*, *Tnf-α*, *Prok2*, *Scn11a*, and *Trpa1*, among others) with significantly increased expression in MOR-tolerant rats compared to the saline control. Those genes are correlated with the upregulation of pain and related pathways, including inflammation, eicosanoid metabolism, and sodium/ion channels. Concomitantly in MOR rats, we found increased expression of genes (*Cnr2*, *Gdnf*, *Il-10*) that were correlated with the decrease of pain. Under melatonin coinfusion (3 μg/h, i.t.), MOR + Mela rats showed only one significantly increased gene, *Kcnip3* (Potassium Voltage-Gated Channel Interacting Protein 3, also known as downstream regulatory element antagonist modulator (DREAM)).

**Figure 5:**
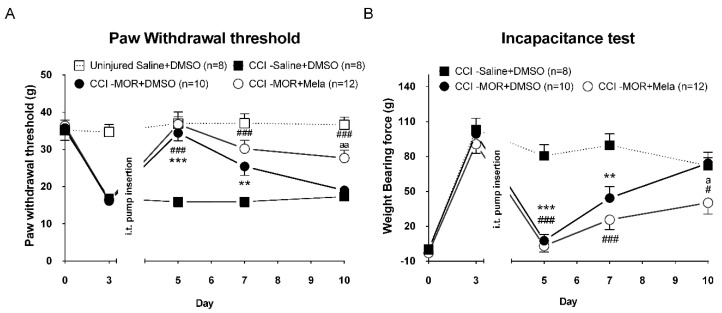
Normal saline + DMSO, MOR (15 μg/h) + DMSO, or MOR + Mela (3 μg/h) was administrated via i.t. infusion to CCI rats in a normal LD cycle room. The paw withdrawal threshold (**A**) and weight-bearing test (**B**) were measured before CCI surgery as a baseline and at day 3 post-CCI as the treatment baseline. After osmotic pump implantation at day 4 post-CCI, the behavior tests were measured at days 5, 7, and 10 post-CCI surgery. * denotes statistically significant differences between CCI-MOR + DMSO and CCI-saline + DMSO. # denotes statistically significant differences between CCI-MOR + Mela and CCI-saline + DMSO. “a” denotes statistically significant differences between CCI-MOR + Mela and CCI-MOR + DMSO. #/a *p* < 0.05; **/aa *p* < 0.05; ***/### *p* < 0.001.

To determine whether the exogenous melatonin coinfusion with morphine was similarly functional in the neuropathic pain model, we employed a rat model of CCI-induced neuropathic pain. All treated groups showed a significant reduction of the paw withdrawal threshold (Figure 5A) and increased asymmetry differences of the weight-bearing (Figure 5B) at day 3 post-CCI surgery. Four days later, the i.t. pump with saline + DMSO, MOR (15 μg/h) + DMSO or MOR (15 μg/h) + Mela (3 μg/h) was implanted in the CCI rats. Both MOR + DMSO and MOR + Mela infusions inhibited the nociception response on the first day of infusion. The morphine’s antinociceptive effect was decreasing gradually toward day 10 post-CCI, suggesting a significant tolerance development. At the same time, MOR + Mela coinfused CCI rats showed only a partial tolerance (Figure 5A, CCI-MOR + Mela: 27.7 ± 2.1 g and Figure 5B, CCI-MOR + Mela: Δforce 40.1 ± 9.7 g) at the end of the experiment, with a significant higher antinociceptive effect compared to MOR alone (Figure 5A, CCI-MOR + DMSO: 19.0 ± 1.2 g and Figure 5B, CCI-MOR + DMSO: Δforce 74.1 ± 9.4 g).

## 4. Discussion

In this study, a disrupted circadian light pattern was introduced to neuropathic rats to mimic light pollution and alter the animals’ internal circadian physiology. Under constant light exposure, the neuropathic rats exhibited an increased morphine tolerance compared to animals housed in the regular light cycle, indicating the impact of the constant light exposure on pain modulation. Moreover, we tested on both naïve and neuropathic rats exogenous melatonin (a circadian hormone) coadministration, which significantly reduced morphine tolerance while maintaining a conserved morphine sensitivity relative to control-tolerant rats. Following long-term i.t. infusion, this low-dose melatonin augmented the antioxidative enzymes (SOD2, HO-1, and GPx1) in the spinal cord of morphine-tolerant rats, which manifested in a receptor-independent mechanism as shown by the absence of effect of MT receptor inhibitors (luzindole and 4P-PDOT) in the reversal of melatonin’s effect. At the transcriptional level, we found numerous proinflammatory and pain-related receptor genes significantly upregulated in morphine-tolerant rats. In contrast, the rats coinfused with low-dose melatonin, without the significant inflammatory gene expression, solely showed the significantly increased gene expression of *Kcnip3*, which has been linked to reduced sensitivity of inflammatory pain in rats.

In a previous report, constant light exposure (but not constant dark) abolished the nocturnal peak of melatonin secretion, along with increased irritability and reduced overall activity [76]. Moreover, sleep deprivation was found to suppress melatonin secretion and enhance pain behavior in neuropathic pain rats, which were significantly ameliorated by daily melatonin supplementation [24]. More importantly, rats with morphine-induced hyperalgesia and tolerance exhibited a significantly lower serum melatonin level and reduction in expression of the MT1 receptors [36], which implied complicated interactions between the circadian hormone and morphine tolerance. Our animal experiments were based on the CCI of the sciatic nerve with an i.t. morphine-tolerance model; it indicates that long-term dysregulated light exposure could jeopardize morphine sensitivity and accelerate tolerance development, which could be a result of the abolished melatonin oscillation in neuropathic pain.

Our evidence also shows that continued/low concentration (3 μg/h, i.t.) melatonin coinfusion with morphine could sufficiently reduce tolerance and partially restore morphine sensitivity in both naïve and neuropathic rats; meanwhile, at a higher dose (6 μg/h, i.t.), has no impact on the tolerance. This differential effect could be related to the potential physiological level of melatonin. In accordance with our findings, Irina et al. observed that lower-dose melatonin (0.3 mg, p.o.) was sufficient to restore the nocturnal physiological level of melatonin in insomnia patients; it also provided the most significant effect on sleep improvement compared to a pharmacological dose (3 mg, p.o.) [77]. Similarly, Lewy et al. reported that lower doses of melatonin, 20–300 μg, were sufficient to reach the physiological level and synchronize the free-running circadian rhythm in blind subjects [78]. Our i.t. injection model bypassed the liver metabolism and mimicked the circulating melatonin in the cerebrospinal fluid (CSF), suppressing morphine tolerance. The finding suggested that low-dose melatonin could be coinfused with morphine in clinical i.t. injection or patient-controlled analgesia for pain management. The concentration reached by continued i.t. infusion, as well as the physiological and therapeutic concentration of melatonin in CSF, remain to be explored.

It is worth noting that most of the antinociceptive effects induced by melatonin were tested with a higher dosage (up to 300 mg, p.o.), and their mechanisms of action were correlated with MT2 receptors or opioid receptors [79,80,81]. For instance, Ambriz-Tututi and Granados-Soto demonstrated that intrathecal (up to 100 μg), and oral (37.5–300 mg/kg) administration of melatonin decreased tactile allodynia in rats with spinal nerve ligation, with the involvement of both MT2 and opioid receptors [82]. Lin et al. showed that administration of 100 mg/kg (i.p.) melatonin attenuated both thermal and mechanical allodynia, and suggested an involvement of both MT2-dependent and -independent pathways in sciatic nerve cuff-implanted mice [83]. However, our long-term and low-dose melatonin administration exhibited no antinociceptive effect on its own, but attenuated morphine tolerance in a melatonin receptor-independent pathway, as shown by the similar findings of a metabolite AFMK cotreatment on morphine tolerance and the ineffective blockage of MT-receptor inhibitors. Consistent with our results, a recent report showed that low-dose melatonin (i.p. injection, 0.5 mg/kg daily) rescued morphine tolerance via decreasing NLRP3 inflammasome activation and reactive oxygen species (ROS) levels [84]. Importantly, our finding of the upregulated antioxidative enzymes in MOR + Mela rats suggested that outcomes resulted from the potent antioxidant properties of melatonin and its metabolites [85].

Importantly, several reports have suggested that melatonin also triggers an antioxidant response via the nuclear factor erythroid 2-related factor 2 and antioxidant responsive element (NRF2-ARE) signaling pathway. Chen et al. reported that daily injection of 10–100 mg/kg melatonin rescued gamma-hydroxybutyric acid intoxication injury via the nuclear translocation of NRF2 and upregulation of SOD1, CAT, and GSH-Px expression [86]. Ali et al. found that a single i.p. injection of 20 mg/kg melatonin prevents acute ethanol-induced neurotoxicity and reverses the oxidative stress via an NRF2-dependent mechanism [87]. Moreover, both melatonin (i.p.) and the synthetic MT1/MT2 receptor agonist Ramelteon (p.o., daily) were used in acute and chronic traumatic brain injury models, respectively [88,89]. Both studies found neuroprotection with a significant increase of antioxidative enzymes, in an NRF2-dependent manner. Additionally, Redondo et al. showed that an NRF2 activator (sulforaphane) increased the local antinociceptive actions of morphine on a complete Freund’s adjuvant-induced pain model [90]. Our findings implied that the chronic infusion of melatonin/AFMK might enhance the NRF2-ARE pathway in the spinal cord. Further dose/time-dependent evaluation on the upstream antioxidative response in analgesic tolerance is necessary.

Our gene expression profiling showed a shift toward pronociceptive pathways in morphine-tolerance with significant upregulation of proinflammatory/ion channel mRNA. Evidence from previous studies has shown a strong correlation of inflammation with morphine tolerance [91]. This imbalance of nociception was down to the basal level upon melatonin cotreatment. The strong interaction of melatonin with the inflammatory pathway was widely discussed [92], and its anti-inflammatory effect explained the attenuation of morphine tolerance development, as we observed in our rat model.

The *Kcnip3* gene encodes a Ca^2+^-binding protein, Kv channel-interacting protein 3 (KChIP3), which functions as a transcriptional repressor, also known as downstream regulatory element antagonist modulator (DREAM). It was first known as a local silencer of the downstream regulatory element (DRE) sequence on the prodynorphin gene [93]. The genetic loss of DREAM in mice showed an attenuated pain behavior via enhanced expression of prodynorphin and dynorphin (an endogenous opioid peptide primarily targeting κ-opiate receptors) in the spinal cord [94]. However, the gain-of-function study in mice suggested basal hyperalgesia via the reduction of prodynorphin and brain-derived neurotrophic factor, while hypoalgesia was found in response to the intraplantar injection of complete Freund’s adjuvant (CFA) [95].

Nonetheless, Zhang et al. reported a neuroprotective effect of DREAM against NMDA-induced toxicity via the inhibition of NMDA receptor-mediated current, specifically through interacting with the NR1 subunit [96]. Furthermore, Tian et al. found that DREAM (N-terminal 31–50 fragment) interacts with transient receptor potential ion channel V1 (TRPV1) and exogeneous DREAM or N-terminal 31–50 fragment peptide alleviated CFA-induced thermal hyperalgesia behavior [65]. On the other hand, the genetic loss of DREAM in rats showed enhanced pain sensitivity in the CFA-induced pain model, along with increased anxiety/depression-like behavior [64,65]. Consistent with our findings, the alleviated morphine tolerance in melatonin-cotreated rats may be attributed to the increased *Kcnip3* expression. In fact, the DREAM protein shares many characteristics with melatonin. The DREAM protein has circadian oscillation in both the pineal gland and retina, and it also binds to the DRE-site of the aralkylamine N-acetyltransferase, the enzyme involved in melatonin synthesis [97]. Moreover, Rivas et al. demonstrated the protection of DREAM against oxidative stress based on the PC12 cell model [98]. Our gene expression profiling suggests a potential inter-regulation between melatonin and DREAM-mediated regulation. Their dynamic interplay in the modulation of pain, anxiety, and sleep could converge and participate in multiple components of pain. A particular focus with multiple experimental approaches on this interaction is necessary.

The potential use of melatonin for pain management has been extensively studied [99]. Further evidence has suggested other possibilities in the use of melatonin as an adjuvant for chemotherapy, metabolic disorders, and fibromyalgia [100,101,102,103]. This further indicates the importance of regular circadian rhythms and the multitargeting potential of exogenous melatonin.

## 5. Conclusions

Opioids have long been useful for chronic pain management, and their use is highly prevalent among adults with severe pain interference (up to 69% in 2013–2014) who use any prescription drug for pain management in the United States [104]. However, tolerance demands a clinical dose escalation, which further increases the risk of overdose and substance use disorder [105]. Our study suggests that a proper circadian light control in the hospital ward could help to entrain patients’ normal circadian rhythms and prevent the exacerbation of morphine tolerance, and that a long-term coadministration of low-dose melatonin with morphine could be a feasible, effective, and physiological adjuvant for chronic opioid users to avoid dose-escalation, dependence, and overdose.

## Figures and Tables

**Table 1:** Gene expression array of rats treated with an i.t. pump of MOR (15 μg/h) alone or combined with 3 μg/h melatonin (MOR + Mela). Fold changes are shown as compared to the saline-treated control.

Genes	MOR vs. Saline Control			MOR + Mela vs. Saline Control						
	Fold Regulation	95% CI	*p*-Value	Fold Regulation	95% CI	*p*-Value	Pathway	↑ Pain	↓ Pain	Ref.
*Alox5*	1.637	(1.29, 1.98)	* 0.0151	1.082	(0.88, 1.29)	0.4274	Inflammation	■		[48,49,50]
*Ccr2*	4.916	(0.00001, 9.90)	* 0.0239	1.603	(0.00001, 3.30)	0.6084	Inflammation	■		[51,52,53]
*Cnr2*	1.867	(1.25, 2.49)	* 0.0204	1.399	(0.75, 2.05)	0.2291	Cannabinoid receptors		■	[54,55]
*Gdnf*	2.001	(1.08, 2.92)	0.0768	1.067	(0.73, 1.40)	0.7764	Neurotrophin		■	[56,57]
*Il10*	6.324	(1.49, 11.16)	* 0.0112	1.165	(0.38, 1.95)	0.8258	Inflammation		■	[58,59]
*Il1a*	1.545	(1.10, 1.99)	* 0.0353	−1.172	(0.42, 1.28)	0.6572	Inflammation	■		[60,61,62]
*Il6*	3.177	(0.90, 5.45)	* 0.0191	1.354	(0.41, 2.30)	0.5021	Inflammation	■		[53,63]
*Kcnip3*	1.036	(0.85, 1.22)	0.7624	1.342	(1.07, 1.62)	*0.0418	Potassium channel		■	[64,65]
*Pla2g1b*	1.637	(1.21, 2.06)	* 0.0399	−1.219	(0.47, 1.17)	0.5058	Eicosanoid metabolism	■		[66]
*Prok2*	3.920	(0.97, 6.87)	*0.0333	1.455	(0.62, 2.29)	0.2562	Inflammation	■		[67,68]
*Ptger3*	1.974	(1.09, 2.86)	* 0.0493	1.339	(0.74, 1.94)	0.2369	Eicosanoid metabolism	■		[69,70]
*Ptger4*	1.330	(1.09, 1.57)	* 0.0366	1.246	(0.98, 1.51)	0.1199	Eicosanoid metabolism	■		[71]
*Scn11a*	4.996	(2.38, 7.61)	* 0.0203	1.072	(0.79, 1.35)	0.6871	Sodium channel	■		[72]
*Tnf*	2.923	(0.87, 4.97)	* 0.0328	1.702	(0.47, 2.93)	0.2336	Inflammation	■		[53,63]
*Trpa1*	3.366	(1.96, 4.77)	* 0.0184	1.182	(0.95, 1.41)	0.1638	Ion channel	■		[73,74]
*Trpv1*	1.415	(1.10, 1.73)	* 0.0400	1.048	(0.79, 1.31)	0.7101	Ion channel	■		[73,74,75]

Spinal cord cDNA samples of saline-DMSO- (*n* = 7), MOR + DMSO- (*n* = 8), and MOR + Mela (*n* = 7) -treated rats were randomly pooled and analyzed using three independent measurements of the RT^2^ Profiler PCR Array. * denotes statistically significant differences between MOR + Mela or MOR and the saline control.

## Supplementary Materials

The following are available online at https://www.mdpi.com/2076-3921/9/9/780/s1, Figure S1: Dose-dependent effect of i.t. melatonin on the Wistar rat morphine tolerance development. Figure S2: Mouse microglia ECO 13.31 cells were incubated with morphine (50–200 μM) alone or cotreated with 20–50 μM of melatonin for 72 h. Table S1: Full gene expression array of rats treated with an i.t. pump of MOR (15 μg/h) alone or combined with 3 μg/h melatonin (MOR + Mela). Table S2: The table of gene expression array with Refseq no. and description.
