# Supplementary material for: The Circadian Hormone Melatonin Inhibits Morphine-Induced Tolerance and Inflammation via the Activation of Antioxidative Enzymes
Source: Antioxidants (Basel). 2020 Aug 22;9(9):780. doi: 10.3390/antiox9090780 (PMC7555201; doi:10.3390/antiox9090780)
Supplement: Supplementary file 1 [file antioxidants-09-00780-s001.pdf]

Supplementary Materials

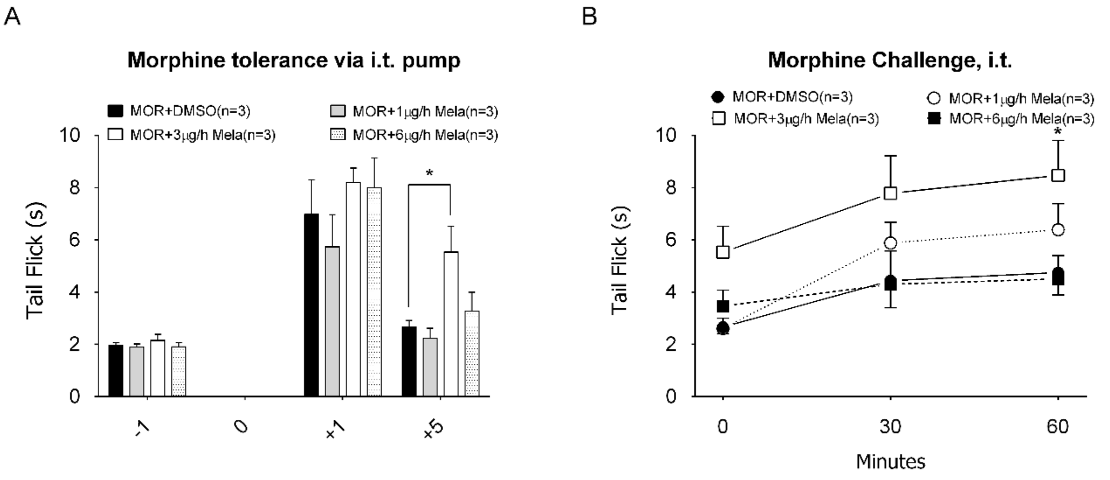

Figure S1. Dose-dependent effect of i.t. melatonin on the Wistar rat morphine tolerance development. (**A**) Continuous administration of Mela (1, 3, or 6 μg/h) or solvent-DMSO in morphine (MOR)-induced tolerant rats via i.t. pump infusion. Tail-flick test was performed before and 1 and 5 days after pump implantation (day 0) for morphine tolerance measurement. (**B**) After i.t. pump infusion for five days, rats were further challenged with 15 μg of morphine (i.t.). Tail-flick test was performed for 120 min to validate the tolerance. * denotes statistically significant differences between MOR + Mela and MOR + DMSO. * $p < 0.05$.

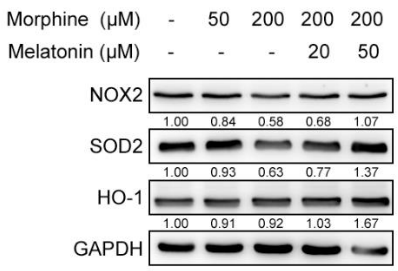

Figure S2. Mouse microglia ECO 13.31 cells were incubated with morphine (50–200 μM) alone or cotreated with 20–50 μM of melatonin for 72 h. Total protein lysates were collected, and the relative protein expression level of NOX2, SOD2, and HO-1 were determined by immunoblotting with respective antibodies.

Table S1. Full gene expression array of rats treated with an i.t. pump of MOR (15 μg/h) alone or combined with 3 μg/h melatonin (MOR + Mela). Fold changes are shown as compared to the saline-treated control.

Genes	MOR vs Control			MOR + Mela vs Control			Pathway
	**Fold Regulation**	**95% CI**	***p*-Value**	**Fold Regulation**	**95% CI**	***p*-Value**	
Alox5	1.637	(1.29, 1.98)	* 0.0151	1.082	(0.88, 1.29)	0.4274	Inflammation
Bdnf	1.412	(0.67, 2.16)	0.2630	1.425	(0.62, 2.23)	0.2536	Neurotrophin
Calca	−1.148	(0.20, 1.55)	0.5764	2.441	(0.35, 4.53)	0.0965	Inflammation
Cckbr	1.238	(0.83, 1.65)	0.2556	1.517	(0.83, 2.20)	0.1320	Inflammation
Ccl12	2.709	(0.00001, 8.21)	0.5535	1.808	(0.00001, 5.39)	0.9247	Inflammation
Ccr2	4.916	(0.00001, 9.90)	* 0.0239	1.603	(0.00001, 3.30)	0.6084	Inflammation
Cnr2	1.867	(1.25, 2.49)	* 0.0204	1.399	(0.75, 2.05)	0.2291	Cannabinoid receptors
Cx3cr1	1.351	(0.99, 1.72)	0.1185	1.342	(1.06, 1.62)	0.0570	Inflammation
Edn1	1.698	(0.79, 2.61)	0.0787	1.155	(0.41, 1.90)	0.7042	Inflammation
Gch1	1.418	(0.84, 2.00)	0.1859	1.405	(0.71, 2.10)	0.2835	Inflammation
Gdnf	2.001	(1.08, 2.92)	0.0768	1.067	(0.73, 1.40)	0.7764	Neurotrophin
Grm1	1.144	(0.00001, 3.53)	0.8376	1.644	(0.00001, 4.90)	0.8744	Glutamate receptor
Il10	6.324	(1.49, 11.16)	* 0.0112	1.165	(0.38, 1.95)	0.8258	Inflammation
Il1a	1.545	(1.10, 1.99)	* 0.0353	−1.172	(0.42, 1.28)	0.6572	Inflammation
Il1b	1.859	(0.54, 3.18)	0.1303	1.098	(0.43, 1.76)	0.9540	Inflammation
Il6	3.177	(0.90, 5.45)	* 0.0191	1.354	(0.41, 2.30)	0.5021	Inflammation
Itgb2	1.920	(0.81, 3.03)	0.0978	2.236	(0.00001, 4.94)	0.1793	Inflammation
Kcnip3	1.036	(0.85, 1.22)	0.7624	1.342	(1.07, 1.62)	*0.0418	Potassium channel
Ngf	1.969	(0.64, 3.30)	0.0915	1.038	(0.22, 1.85)	0.9538	Neurotrophin
Ntrk1	2.053	(0.24, 3.86)	0.1517	1.531	(0.45, 2.62)	0.2656	Neurotrophin
Oprm1	1.212	(0.46, 1.96)	0.6298	1.305	(0.34, 2.27)	0.4802	Opioid receptor
P2rx3	1.787	(1.08, 2.49)	0.0565	1.171	(0.73, 1.61)	0.4114	Purinergic receptor
Penk	1.396	(0.97, 1.83)	0.1091	1.024	(0.79, 1.26)	0.9365	Inflammation
Pla2g1b	1.637	(1.21, 2.06)	* 0.0399	−1.219	(0.47, 1.17)	0.5058	Eicosanoid metabolism
Prok2	3.920	(0.97, 6.87)	* 0.0333	1.455	(0.62, 2.29)	0.2562	Inflammation
Ptger3	1.974	(1.09, 2.86)	* 0.0493	1.339	(0.74, 1.94)	0.2369	Eicosanoid metabolism
Ptger4	1.330	(1.09, 1.57)	* 0.0366	1.246	(0.98, 1.51)	0.1199	Eicosanoid metabolism
Ptgs1	1.345	(0.00001, 3.66)	0.8421	−2.128	(0.00001, 1.39)	0.4535	Eicosanoid metabolism
Ptgs2	1.552	(0.51, 2.59)	0.2465	1.048	(0.27, 1.83)	0.9605	Eicosanoid metabolism
Scn10a	1.938	(0.71, 3.16)	0.1186	1.184	(0.28, 2.09)	0.7510	Sodium channel
Scn11a	4.996	(2.38, 7.61)	* 0.0203	1.072	(0.79, 1.35)	0.6871	Sodium channel
Tnf	2.923	(0.87, 4.97)	* 0.0328	1.702	(0.47, 2.93)	0.2336	Inflammation
Trpa1	3.366	(1.96, 4.77)	* 0.0184	1.182	(0.95, 1.41)	0.1638	Ion channel
Trpv1	1.415	(1.10, 1.73)	* 0.0400	1.048	(0.79, 1.31)	0.7101	Ion channel
Trpv3	−1.076	(0.00001, 2.28)	0.9723	−1.658	(0.00001, 1.46)	0.5509	Ion channel

Spinal cord cDNA samples of saline-DMSO (n = 7), MOR + DMSO (n = 8), and MOR + Mela (n = 7) -treated rats were randomly pooled and analyzed using three independent measurements of the RT^2 Profiler PCR Array.

Table S2. The table of gene expression array with Refseq no. and description.

PCR Array Catalog #PARN-162Z				
**Position**	**Refseq**	**Symbol**	**Description**	**Gene Name**
A01	NM_012544	Ace	Angiotensin I-converting enzyme (peptidyl-dipeptidase A) 1	Dcp1/StsRR92
A02	NM_017155	Adora1	Adenosine A1 receptor	-
A03	NM_012492	Adrb2	Adrenergic, beta-2-, receptor, surface	-
A04	NM_012822	Alox5	Arachidonate 5-lipoxygenase	LOX5A
A05	NM_030851	Bdkrb1	Bradykinin receptor B1	BKR/Bdkrb/b1bkr
A06	NM_012513	Bdnf	Brain-derived neurotrophic factor	-
A07	NM_147141	Cacna1b	Calcium channel, voltage-dependent, N type, alpha 1B subunit	BIII
A08	NM_017338	Calca	Calcitonin-related polypeptide alpha	CAL6/CGRP/Cal1/Calc/RATCAL6/calcitonin
A09	NM_012829	Cck	Cholecystokinin	-
A10	NM_013165	Cckbr	Cholecystokinin B receptor	Cck2r/Cholrec
A11	NM_001105822	Ccl12	Chemokine (C-C motif) ligand 12	MCP-5
A12	NM_021866	Ccr2	Chemokine (C-C motif) receptor 2	-
B01	NM_031518	Cd200	Cd200 molecule	Cspmo2/MRCOX2/Mox2
B02	NM_012705	Cd4	Cd4 molecule	W3/25/p55
B03	NM_024354	Chrna4	Cholinergic receptor, nicotinic, alpha 4	NARAC
B04	NM_012784	Cnr1	Cannabinoid receptor 1 (brain)	SKR6R
B05	NM_020543	Cnr2	Cannabinoid receptor 2 (macrophage)	CB-2/CB2/CB2C/CNR2C
B06	NM_012531	Comt	Catechol-O-methyltransferase	-
B07	NM_023981	Csf1	Colony-stimulating factor 1 (macrophage)	-
B08	NM_133534	Cx3cr1	Chemokine (C-X3-C motif) receptor 1	Rbs11
B09	NM_013158	Dbh	Dopamine beta-hydroxylase (dopamine beta-monooxygenase)	DOPBHY
B10	NM_012548	Edn1	Endothelin 1	Et1
B11	NM_012550	Ednra	Endothelin receptor type A	ET-A/ET-AR/Endor/Eta/RGD1559432
B12	NM_024132	Faah	Fatty acid amide hydrolase	-
C01	NM_024356	Gch1	GTP cyclohydrolase 1	Gch
C02	NM_019139	Gdnf	Glial cell-derived neurotrophic factor	gndf

C03	NM_017010	Grin1	Glutamate receptor, ionotropic, N-methyl D-aspartate 1	GluN1/NMDAR1/NR1
C04	NM_012574	Grin2b	Glutamate receptor, ionotropic, N-methyl D-aspartate 2B	GluN2B
C05	NM_017011	Grm1	Glutamate receptor, metabotropic 1	Gprc1a
C06	NM_017012	Grm5	Glutamate receptor, metabotropic 5	mGluR5/mGlur5
C07	NM_012585	Htr1a	5-hydroxytryptamine (serotonin) receptor 1A	5HT1A/RAT5HT1A
C08	NM_017254	Htr2a	5-hydroxytryptamine (serotonin) receptor 2A	5-HT2A/5Ht-2
C09	NM_012854	Il10	Interleukin 10	IL10X
C10	NM_019165	Il18	Interleukin 18	IL-18
C11	NM_017019	Il1a	Interleukin 1 alpha	IL-1 alpha
C12	NM_031512	Il1b	Interleukin 1 beta	-
D01	NM_053836	Il2	Interleukin 2	-
D02	NM_012589	Il6	Interleukin 6	ILg6/Ifnb2
D03	NM_012711	Itgam	Integrin, alpha M	Cd11b
D04	NM_001037780	Itgb2	Integrin, beta 2	Cd18
D05	NM_032462	Kcnip3	Kv channel interacting protein 3, calsenilin	Csen/Dream/rKChIP3
D06	NM_013192	Kcnj6	Potassium inwardly rectifying channel, subfamily J, member 6	-
D07	NM_133322	Kcnq2	Potassium voltage-gated channel, KQT-like subfamily, member 2	-
D08	NM_031597	Kcnq3	Potassium voltage-gated channel, KQT-like subfamily, member 3	-
D09	NM_013198	Maob	Monoamine oxidase B	-
D10	NM_053842	Mapk1	Mitogen-activated protein kinase 1	ERK-2/ERT1/Erk2/p42-MAPK
D11	NM_031020	Mapk14	Mitogen-activated protein kinase 14	CRK1/CSBP/CSPB1/Csbp1/Csbp2/Exip/Hog/Mxi2/Prkm14/Prkm15/RK/Sapk2A/p38/p38Hog/p38alpha
D12	NM_017347	Mapk3	Mitogen-activated protein kinase 3	ERK1/ERT2/Erk-1/Esrk1/MAPK1/MNK1/Prkm3/p44/p44erk1/p44mapk

E01	NM_053829	Mapk8	Mitogen-activated protein kinase 8	JNK
E02	NM_001277055	Ngf	Nerve growth factor (beta polypeptide)	Ngfb/beta-NGF
E03	NM_021589	Ntrk1	Neurotrophic tyrosine kinase, receptor, type 1	Trk
E04	NM_012617	Oprd1	Opioid receptor, delta 1	-
E05	NM_017167	Oprk1	Opioid receptor, kappa 1	-
E06	NM_013071	Oprm1	Opioid receptor, mu 1	MORA/Oprm/Oprrm1
E07	NM_031075	P2rx3	Purinergic receptor P2X, ligand-gated ion channel, 3	-
E08	NM_031594	P2rx4	Purinergic receptor P2X, ligand-gated ion channel 4	-
E09	NM_019256	P2rx7	Purinergic receptor P2X, ligand-gated ion channel, 7	-
E10	NM_012800	P2ry1	Purinergic receptor P2Y, G-protein coupled, 1	P2y/P2y1
E11	NM_019374	Pdyn	Prodynorphin	-
E12	NM_017139	Penk	Proenkephalin	Enk/Penk-rs/Penk1/Penk2
F01	NM_031585	Pla2g1b	Phospholipase A2, group IB, pancreas	-
F02	NM_013007	Pnoc	Prepronociceptin	N23K/Npnc1
F03	NM_138852	Prok2	Prokineticin 2	Bv8
F04	NM_013100	Ptger1	Prostaglandin E receptor 1 (subtype EP1)	EP1
F05	NM_012704	Ptger3	Prostaglandin E receptor 3 (subtype EP3)	EP3/EP3R/Rep3/rEP3a/rEP3b
F06	NM_032076	Ptger4	Prostaglandin E receptor 4 (subtype EP4)	EP4/Ptger/Ptgerep4
F07	NM_021583	Ptges	Prostaglandin E synthase	Pges
F08	NM_001107832	Ptges2	Prostaglandin E synthase 2	-
F09	NM_001130989	Ptges3	Prostaglandin E synthase 3 (cytosolic)	RGD1561913
F10	NM_017043	Ptgs1	Prostaglandin-endoperoxide synthase 1	Cox-1/Cox-3/Cox1/Cox3/Pghs-1
F11	NM_017232	Ptgs2	Prostaglandin-endoperoxide synthase 2	COX-2/Cox2
F12	NM_017247	Scn10a	Sodium channel, voltage-gated, type X, alpha subunit	Na(V)1.8/Nav1.8/PN3
G01	NM_019265	Scn11a	Sodium channel, voltage-gated, type XI, alpha	NaN
G02	NM_013119	Scn3a	Sodium channel, voltage-gated, type III, alpha	Nav1.3/SCIII/Scn2a

G03	NM_133289	Scn9a	Sodium channel, voltage-gated, type IX, alpha	Nav1.7/PN1/Scn2a
G04	NM_031343	Slc6a2	Solute carrier family 6 (neurotransmitter transporter, noradrenalin), member 2	Net
G05	NM_012666	Tac1	Tachykinin 1	PPTA3/Ppt5fl/RATPPTA3/TAC
G06	NM_012667	Tacr1	Tachykinin receptor 1	Tac1r
G07	NM_198769	Tlr2	Toll-like receptor 2	-
G08	NM_019178	Tlr4	Toll-like receptor 4	-
G09	NM_012675	Tnf	Tumor necrosis factor (TNF superfamily, member 2)	RATTNF/TNF-alpha/Tnfa
G10	NM_207608	Trpa1	Transient receptor potential cation channel, subfamily A, member 1	Anktm1
G11	NM_031982	Trpv1	Transient receptor potential cation channel, subfamily V, member 1	TRPV1_SON/VR.5'sv/Vr1/Vr1l1
G12	NM_001025757	Trpv3	Transient receptor potential cation channel, subfamily V, member 3	-
H01	NM_031144	Actb	Actin, beta	Actx
H02	NM_012512	B2m	Beta-2 microglobulin	-
H03	NM_012583	Hprt1	Hypoxanthine phosphoribosyltransferase 1	Hgprtase/Hprt
H04	NM_017025	Ldha	Lactate dehydrogenase A	Ldh1
H05	NM_001007604	Rplp1	Ribosomal protein, large, P1	-
H06	U26919	RGDC	Rat genomic DNA contamination	RGDC
H07	SA_00104	RTC	Reverse transcription control	RTC
H08	SA_00104	RTC	Reverse transcription control	RTC
H09	SA_00104	RTC	Reverse transcription control	RTC
H10	SA_00103	PPC	Positive PCR control	PPC
H11	SA_00103	PPC	Positive PCR control	PPC
H12	SA_00103	PPC	Positive PCR control	PPC
